# Supplementary material for: HIV prevalence, risk factors, prevention methods, and interventions among taxi drivers and commercial motorcyclists in sub-Saharan Africa: A scoping review
Source: PLOS Glob Public Health. 2025 May 29;5(5):e0004239. doi: 10.1371/journal.pgph.0004239 (PMC12121784; doi:10.1371/journal.pgph.0004239)
Supplement: S1 Table — (DOCX) [file pgph.0004239.s001.docx]

**Supplementary file 1.** Search strategy for article selection (PUBMED)

| **Search** | **Query** |
| --- | --- |
| #6 | (((((Taxi drivers[Title/Abstract] OR motor vehicle taxi drivers[Title/Abstract] OR minibus taxi drivers[Title/Abstract] OR minibus drivers[Title/Abstract] OR Boda Boda riders[Title/Abstract] OR Boda Boda operators[Title/Abstract] OR Boda Boda motorcycle drivers[Title/Abstract] OR Boda Boda motorcyclists[Title/Abstract] OR motorcycle drivers[Title/Abstract] OR commercial motorcycle drivers[Title/Abstract] OR tricycle drivers[Title/Abstract]) AND (HIV[Title/Abstract] OR acquired immune syndrome[Title/Abstract] OR HIV-2[Title/Abstract] OR HIV-1[Title/Abstract] OR HIV infections[Title/Abstract])) AND (Prevalence OR frequency OR extent OR incidence OR rate OR proportion OR percent* OR seroprevalence OR HIV serodiagnosis OR HIV seropositive* OR HIV seronegative* or HIV test*)) AND (Predictors OR associated factors OR correlates OR risk factors OR risky sexual behaviors OR unsafe sex OR risk taking OR health risk behaviors OR sexual behaviors)) AND (Condoms OR condom use OR condom programming OR HIV testing OR AIDS testing OR voluntary counsel* OR self-testing OR diagnosis OR point-of-care testing OR mass screening OR HIV testing and counsel* OR serologic test OR pre-exposure prophylaxis OR PREP OR antiretroviral agents OR antiretroviral therapy OR HIV infections/drug therapy OR highly active OR anti-HIV agents OR circumcision OR safe male circumcision OR male circumcision OR SMC OR use OR utilize*)) AND (Intervention OR strategy* OR plans OR strategic plans OR improve* OR advances OR policy* OR approach*) |
| #5 | ((((Taxi drivers[Title/Abstract] OR motor vehicle taxi drivers[Title/Abstract] OR minibus taxi drivers[Title/Abstract] OR minibus drivers[Title/Abstract] OR Boda Boda riders[Title/Abstract] OR Boda Boda operators[Title/Abstract] OR Boda Boda motorcycle drivers[Title/Abstract] OR Boda Boda motorcyclists[Title/Abstract] OR motorcycle drivers[Title/Abstract] OR commercial motorcycle drivers[Title/Abstract] OR tricycle drivers[Title/Abstract]) AND (HIV[Title/Abstract] OR acquired immune syndrome[Title/Abstract] OR HIV-2[Title/Abstract] OR HIV-1[Title/Abstract] OR HIV infections[Title/Abstract])) AND (Prevalence OR frequency OR extent OR incidence OR rate OR proportion OR percent* OR seroprevalence OR HIV serodiagnosis OR HIV seropositive* OR HIV seronegative* or HIV test*)) AND (Predictors OR associated factors OR correlates OR risk factors OR risky sexual behaviors OR unsafe sex OR risk taking OR health risk behaviors OR sexual behaviors)) AND (Condoms OR condom use OR condom programming OR HIV testing OR AIDS testing OR voluntary counsel* OR self-testing OR diagnosis OR point-of-care testing OR mass screening OR HIV testing and counsel* OR serologic test OR pre-exposure prophylaxis OR PREP OR antiretroviral agents OR antiretroviral therapy OR HIV infections/drug therapy OR highly active OR anti-HIV agents OR circumcision OR safe male circumcision OR male circumcision OR SMC OR use OR utilize*) |
| #4 | (((Taxi drivers[Title/Abstract] OR motor vehicle taxi drivers[Title/Abstract] OR minibus taxi drivers[Title/Abstract] OR minibus drivers[Title/Abstract] OR Boda Boda riders[Title/Abstract] OR Boda Boda operators[Title/Abstract] OR Boda Boda motorcycle drivers[Title/Abstract] OR Boda Boda motorcyclists[Title/Abstract] OR motorcycle drivers[Title/Abstract] OR commercial motorcycle drivers[Title/Abstract] OR tricycle drivers[Title/Abstract]) AND (HIV[Title/Abstract] OR acquired immune syndrome[Title/Abstract] OR HIV-2[Title/Abstract] OR HIV-1[Title/Abstract] OR HIV infections[Title/Abstract])) AND (Prevalence OR frequency OR extent OR rate OR proportion OR percent* OR seroprevalence OR HIV serodiagnosis OR HIV seropositive* OR HIV seronegative* or HIV test*)) AND (Predictors OR associated factors OR correlates OR risk factors OR risky sexual behaviors OR unsafe sex OR risk taking OR health risk behaviors OR sexual behaviors) |
| #3 | ((Taxi drivers[Title/Abstract] OR motor vehicle taxi drivers[Title/Abstract] OR minibus taxi drivers[Title/Abstract] OR minibus drivers[Title/Abstract] OR Boda Boda riders[Title/Abstract] OR Boda Boda operators[Title/Abstract] OR Boda Boda motorcycle drivers[Title/Abstract] OR Boda Boda motorcyclists[Title/Abstract] OR motorcycle drivers[Title/Abstract] OR commercial motorcycle drivers[Title/Abstract] OR tricycle drivers[Title/Abstract]) AND (HIV[Title/Abstract] OR acquired immune syndrome[Title/Abstract] OR HIV-2[Title/Abstract] OR HIV-1[Title/Abstract] OR HIV infections[Title/Abstract])) AND (Prevalence OR frequency OR extent OR incidence OR rate OR proportion OR percent* OR seroprevalence OR HIV serodiagnosis OR HIV seropositive* OR HIV seronegative* or HIV test*) |
| #2 | (Taxi drivers[Title/Abstract] OR motor vehicle taxi drivers[Title/Abstract] OR minibus taxi drivers[Title/Abstract] OR minibus drivers[Title/Abstract] OR Boda Boda riders[Title/Abstract] OR Boda Boda operators[Title/Abstract] OR Boda Boda motorcycle drivers[Title/Abstract] OR Boda Boda motorcyclists[Title/Abstract] OR motorcycle drivers[Title/Abstract] OR commercial motorcycle drivers[Title/Abstract] OR tricycle drivers[Title/Abstract]) AND (HIV[Title/Abstract] OR acquired immune syndrome[Title/Abstract] OR HIV-2[Title/Abstract] OR HIV-1[Title/Abstract] OR HIV infections[Title/Abstract]) |
| #1 | Taxi drivers[Title/Abstract] OR motor vehicle taxi drivers[Title/Abstract] OR minibus taxi drivers[Title/Abstract] OR minibus drivers[Title/Abstract] OR Boda Boda riders[Title/Abstract] OR Boda Boda operators[Title/Abstract] OR Boda Boda motorcycle drivers[Title/Abstract] OR Boda Boda motorcyclists[Title/Abstract] OR motorcycle drivers[Title/Abstract] OR commercial motorcycle drivers[Title/Abstract] OR tricycle drivers[Title/Abstract] |
