# Supplementary material for: HIV prevalence, risk factors, prevention methods, and interventions among taxi drivers and commercial motorcyclists in sub-Saharan Africa: A scoping review
Source: PLOS Glob Public Health. 2025 May 29;5(5):e0004239. doi: 10.1371/journal.pgph.0004239 (PMC12121784; doi:10.1371/journal.pgph.0004239)
Supplement: S2 Table — (DOCX) [file pgph.0004239.s002.docx]

**Supplementary file 2: Data extraction form-summary**

| **Thematic Area/subheadings** | **Extracted data** |
| --- | --- |
| Article # |  |
| Full bibliography: | Author surname and study year |
| Title | Study Title |
| Study aim | Study purpose |
| Country of origin/ region | Country e.g. Uganda. Regions included (1= East Africa, 2= Central Africa, 3= West Africa, 4= North Africa, 5= South) |
| Literature type | e.g., Primary research: peer-reviewed research articles  • Epidemiology: articles that have used population-level datasets  • Evidence syntheses: narrative reviews, systematic reviews, scoping reviews, rapid reviews,  etc.  • Conference abstracts: abstracts presented within conferences  • Discussion articles  • Editorials |
| Key themes or Topics discussed by the articles | Key themes or concepts reported in the study e.g prevalence, incidence, associated risk factors, HIV interventions, HIV prevention services etc. |
| Methods and materials: | Study design, settings, study duration (months), population, participants, sample size, study tools, theoretical/conceptual framework etc. |
| Results per thematic area | Findings e.g., for prevalence, incidence, associated risk factors, HIV interventions, HIV prevention services etc. |
| Limitations: | Study limitations in terms of design, sampling, size of the study, setting or data type etc. |
| Conclusion: | Conclusions about the extracted study data in relation to review objectives. |
| Gaps | Questions the study fails to answer. |
| Recommendations | Key recommendations of the study in terms of policy, research, and practice |
