# Supplementary material for: HIV prevalence, risk factors, prevention methods, and interventions among taxi drivers and commercial motorcyclists in sub-Saharan Africa: A scoping review
Source: PLOS Glob Public Health. 2025 May 29;5(5):e0004239. doi: 10.1371/journal.pgph.0004239 (PMC12121784; doi:10.1371/journal.pgph.0004239)
Supplement: Attachment — Submitted filename: Review Prevalence of HIV Taxi Drivers.docx [file pgph.0004239.s004.docx]

Thank you for the opportunity to review the manuscript titled **‘*HIV prevalence, risk factors for infection, use of prevention methods, and interventions among taxi drivers and commercial motorcyclists in sub-Saharan Africa: A scoping review*.’**

Overall, the manuscript is fairly well written. However, I suggest the following comments and clarifications to improve its quality.

1. **Title:** The title is too long**.** I would suggest shortening it to capture the key theme that defines the entire study.
2. **Abstract:**
3. Background: Quote some real figures on HIV prevalence among taxi drivers to show the magnigute of the problem in this population.
4. Methods: Line 41; Change inception to the actual year. The word inception may leave one wondering from when exactly?
5. Results: State the total number of articles initially screened before stating the total number that was finally included.
6. Conclusion: The conclusion seems to deviate from the main objective of the study; state what was the HIV prevalence, the risk factors and prevention methods identified from the current study. What is given is more of implications and recommendations rather than an actual conclusion based on the study objectives and results.
7. **Introduction:**
8. I would suggest introducing the global perspective of HIV before Sub-Saharan Africa in the first paragraph line 70.
9. I would suggest quoting some figures on actual HIV prevalence rates reported in other studies in the first paragraph, to highlight the magnitude of the condition among taxi drivers.
10. A justification on why the study focused only on Sub-Saharan Africa is needed. Taxi drivers in others parts of the world are equally at risk of HIV. A global perspective would have made the evidence produced in this study more stronger.
11. **Methodology:**
12. Line 129: From inception to 2024; State a specific year. ‘Inception’ may not be very clear on which year you are reffering to.
13. Line 167: Cite the SPSS software used in the analysis.
14. Line 168: Elaborate on the qualitative data highlighted here. How did you get these data from the included studies? Did you pick the quotes reported in the studies? The approach used in getting the qualitative data is not very clear.
15. **Results:**
16. Highlight how many studies were identified from the initial search of each individual database.
17. **Discussion**
18. Discuss to acknowledge how the variation in the periods of study publication may have affected the overall quality of your study. Also the Limitations section should take into consideration how studies published in 2005 may differ from those published in 2024, and how this affects the overall evidence reported here.
19. **Conclusuion:**
20. Quote the actual ‘high’ prevalence that you identified from your study. High is relative not very specific. Also, quote the HIV prevalence in the general adult population for a more clear comparison with that of the taxi-drivers.
21. Line 603-604; highlight a few of the behavioral, psychosocial, and sociodemographic risk factors identified in this study.
