# Supplementary material for: HIV prevalence, risk factors, prevention methods, and interventions among taxi drivers and commercial motorcyclists in sub-Saharan Africa: A scoping review
Source: PLOS Glob Public Health. 2025 May 29;5(5):e0004239. doi: 10.1371/journal.pgph.0004239 (PMC12121784; doi:10.1371/journal.pgph.0004239)
Supplement: Attachment — Submitted filename: PGPH-D-25-00089._ReviewerComments.docx [file pgph.0004239.s005.docx]

Dear PLOS Global Public Health Team,

Thank you for the assignment to review the manuscript titled "HIV prevalence, risk factors for infection, use of prevention methods, and interventions among taxi drivers and commercial motorcyclists in sub-Saharan Africa: A scoping review." I appreciate the opportunity to contribute to this important research.

I want to thank the authors for their valuable work. The manuscript touches on a significant public health concern, and the research presented is robust and highly relevant. It addresses essential issues that are crucial for reversing HIV trends in SSA.

Please see my comments below:

1. **Abstract:**

Replace the word “*attributable to”* with “*associated with”* since HIV infection may be associated with psycho*l*ogical issues like stigma but not attributable.

2. **Table 1: Search strategy**

The researcher included the term ***incidence*** and *rate* but the study is reporting prevalence this is confusing, kindly harmonize for clarity. Since you are also mentioning incidence in line 235 I suggest including it in the aim statement besides prevalence

3. **line 136-138:** *This review included studies conducted among taxi drivers and commercial motorcyclists in SSA that reported HIV prevalence, HIV risk factors, use of HIV prevention services, or interventions that directly or indirectly reduced HIV.-*Does this mean that studies where there was no reduction in HIV were excluded?
4. **line 152:** remove the word *by* after the word confirmed to enhance clarity
5. **line 199:** *Nearly half (n=11) were published within the last 5 years*- You would want to specify this period – it may be confusion for audience reading this 10 years later.
6. **lines 199-201:** *Most 200 studies were conducted in the East African region (Figure 2A), with Uganda (n=8) being the largest contributor, followed by Nigeria (n=6) and South Africa (n=5).* This sentence reads as if Uganda , Nigeria and South Africa are all from the East African region.
7. **line 203:** are intervention studies not quantitative. You may want to rephrase this sentence and revise table 2 also.
8. **table 2 :** Nyanzi et al. wrongly listed as qualitative study yet it is mixed methods, Potgieter et al. wrongly listed as qualitative study yet it is quantitative.
9. **line 231:** replace the word *or* with the word *and* then add the word *respectively* after the word partners.
10. **Line 218**: is not concurring with information in figure 2B. Did some studies use more than one method? If so then this should be reported for easier interpretation of figure 2B
11. **Line 312 to 315:** *Notably, no study estimated the magnitude of the risk of HIV associated with drinking alcohol, cigarette smoking, chewing khat, or using other drugs (e.g., chewable tobacco or cocaine) among taxi drivers and commercial motorcyclists.* This sentence contradicts the information provided previously, Furthermore, Challe et al. reported 4.5 times odd disease among those drinking alcohol
