# Supplementary material for: HIV prevalence, risk factors, prevention methods, and interventions among taxi drivers and commercial motorcyclists in sub-Saharan Africa: A scoping review
Source: PLOS Glob Public Health. 2025 May 29;5(5):e0004239. doi: 10.1371/journal.pgph.0004239 (PMC12121784; doi:10.1371/journal.pgph.0004239)
Supplement: Attachment — Submitted filename: Response to Reviewers.doc [file pgph.0004239.s007.doc]

| # | Comment | Response | Page/line number |
| --- | --- | --- | --- |
|  | **Editorial comments** |  |  |
| 1 | 1. Please upload a copy of Figures 1,2,3 which you refer to in your text on page 8, 11, 12. Or, if the figure is no longer to be included as part of the submission please remove all reference to it within the text.    2. Please provide separate figure files in .tif or .eps format.    For more information about figure files please see our guidelines:  LINK  https://journals.plos.org/globalpublichealth/s/figures  https://journals.plos.org/globalpublichealth/s/figures#loc-file-requirements  3. Please review your reference list to ensure that it is complete and correct. If you have cited papers that have been retracted, please include the rationale for doing so in the manuscript text, or remove these references and replace them with relevant current references. Any changes to the reference list should be mentioned in the rebuttal letter that accompanies your revised manuscript. If you need to cite a retracted article, indicate the article's retracted status in the References list and also include a citation and full reference for the retraction notice. | Thanks a lot for the comments. The previous figures have been deleted from the manuscript. New figures have been provided separately in .tif format and uploaded.  All references have been checked, and no study was retracted. |  |
|  | Reviewer #1: |  |  |
| 1 | Line 41 y 129:  I suggest indicating the start date of the search (2000). | Thank you very much for the suggestion. This has been incorporated. The inception period has been noted as January 2000 | Pg 2 LN 41, Pg 5 LN 138 |
| 2 | Line 179:  Study flow: adjust the text corresponding to the articles that have been classified as qualitative research, as of the five articles referenced, the only qualitative one is “Mchunu GG, Naidoo JR, Ncama BP. Condom use: a less travelled route among minibus taxi drivers and their taxi queens in KwaZulu-Natal, South Africa. Afr Health Sci. 2020;20(2):658-65.” | Thank you very much for the suggestion. Although three studies are explicitly qualitative, the other 2 studies were mixed, meaning they had a component of qualitative and quantitative results. Because the PRISMA diagram has no provision for mixed studies, those mixed studies that had some qualitative findings are reported under qualitative synthesis as well as under quantitative synthesis: so we have updated the PRISMA diagram to 21 quantitative (including two intervention studies & two overlapping papers) and 5 qualitative (three purely qualitative studies and two overlapping papers). The following note has been inserted below Figure 1 caption: *“Two studies provided qualitative and quantitative findings (mixed methods) thus have been captured under quantitative and qualitative synthesis. ”*  Studies that were purely qualitative   1. Nyanzi S, Nyanzi B, Kalina B. Contemporary myths, sexuality misconceptions, information sources, and risk perceptions of Bodabodamen in southwest Uganda. Sex Roles. 2005;52:111-9. 2. Mchunu GG, Naidoo JR, Ncama BP. Condom use: a less travelled route among minibus taxi drivers and their taxi queens in KwaZulu-Natal, South Africa. Afr Health Sci. 2020;20(2):658-65 3. Mlughu TS, Anaeli A, Joseph R, Sirili N. Voluntary HIV counseling and testing among commercial motorcyclist youths: an exploration of ethical challenges and coping mechanisms in Dar es Salaam.   722 HIV/AIDS-Research and Palliative Care. 2020:675-85.  Studies that were mixed included:   1. Potgieter C, Strebel A, Shefer T, Wagner C. Taxi 'sugar daddies' and taxi queens: Male taxi driver attitudes regarding transactional relationships in the Western Cape, South Africa. SAHARA-J: Journal of 717 Social Aspects of HIV/AiDS. 2012;9(4):192-9. 2. Tusabe J, Muyinda H, Nangendo J, Kwesiga D, Nabikande S, Muhoozi M, et al. Factors influencing the uptake of voluntary medical male circumcision among Boda-Boda riders aged 18–49 years in Hoima, Western Uganda. HIV/AIDS (Auckland, NZ). 2022;14:437. | Pg 8 LN 203 |
| 3 | Line 195:  Figure 1. Study selection process. Adjust the information related to the “studies included in qualitative synthesis” and “studies included in quantitative synthesis” boxes, as not all the studies have a meta-analysis design. | Thank you very much for the suggestion. Given the explanation in comment #2, we have adjusted figure 1 as suggested. See attached Fig 2 tiff | Pg 8 LN 203 |
| 4 | Line 209:  Table 2. Characteristics of the studies included in this scoping review. Adjust the Study design column for the articles classified as qualitative research, as some are Mixed or Quantitative. | Thank you very much for the suggestion. We rechecked the study design for each of the articles included in this study and have changed these studies to either mixed, quantitative, or qualitative, as deemed necessary. | Pg 9 LN 227 |
| 5 | Line 251:  Figure 3. Conceptual framework. Cite the theoretical sources supporting the proposal | Thank you very much for the suggestion. Although the conceptual framework was formed as a result of the synthesis of all study results, we have cited key theoretical sources supporting the suggested conceptual framework. | Pg 11 LN 270-273 |
| 6 | Line 605:  The phrase “among the study group” - I recommend clarifying that it refers to the articles included in the review. | Thank you very much for the suggestion. This has been changed as suggested | Pg 23 LN 633 |
| 7 | Line 617:  Based on the results presented in this document, review the following conclusion and its scope: “These studies will provide more evidence that may provide comprehensive guidance for better HIV prevention and control policies and programming among taxi drivers and commercial motorcyclists in SSA.” | Thank you very much for the suggestion. We meant that conducting additional research, as suggested in the conclusion, will provide a better picture of HIV programming in this study group. The term “additional studies” has been added to the sentence for clarity, and now it reads: “*These additional studies will provide more evidence that may provide comprehensive guidance for better HIV prevention and control policies and programming among taxi drivers and commercial motorcyclists in SSA*” | Pg 24 LN 645 |
|  | **Reviewer #2:** |  |  |
|  | **Title:** |  |  |
| 1 | The title is too long. I would suggest shortening it to capture the key theme that defines the entire study. | Thank you very much for the suggestion. The title has been shortened to *“HIV prevalence, risk factors, prevention methods, and interventions among taxi drivers and commercial motorcyclists in sub-Saharan Africa: A scoping review”* | Pg 1 LN 1-3 |
|  | **Abstract:** |  |  |
| 2 | Background: Quote some real figures on HIV prevalence among taxi drivers to show the magnitude of the problem in this population. | Thank you very much for the suggestion. Unfortunately, we did not find a single study that solely reports the prevalence of HIV among taxi drivers in SSA. The only study we found aggregated the prevalence of HIV among taxi drivers with that of commercial motorcyclists, which was reported as 33.4%. The other study aggregated the prevalence of HIV among taxi drivers with truck drivers, and this study was not reported (reference #4) | Pg 2 LN 49  Pg 10, LN 251-253 |
| 3 | Methods: Line 41; Change inception to the actual year. The word inception may leave one wondering from when exactly? | Thank you very much for the suggestion. This has been incorporated. The inception period has been noted as January 2000 | Pg 2 LN 41, Pg 5 LN 138 |
| 4 | Results: State the total number of articles initially screened before stating the total number that was finally included. | Thank you very much for the suggestion. The total number of articles initially screened has been added. The sentence has been changed to "*This review included 24 out of 126 studies*.” | Pg 2 LN 48 |
| 5 | Conclusion: The conclusion seems to deviate from the main objective of the study; state what was the HIV prevalence, the risk factors and prevention methods identified from the current study. What is given is more of implications and recommendations rather than an actual conclusion based on the study objectives and results. | Thank you very much for the suggestion. A conclusive sentence related to HIV prevalence, the risk factors, and prevention methods has been added and it reads: "*We found a high HIV prevalence among taxi drivers and commercial motorcyclists in SSA, which may be associated with numerous behavioral, psychosocial, and sociodemographic risk factors. The use of HIV prevention methods such as SMC and condoms was suboptimal, and there were few interventional studies on HIV among the articles included in the review.”* | Pg 2-3, LN 59-70 |
|  | **Introduction:** |  |  |
| 6 | I would suggest introducing the global perspective of HIV before Sub-Saharan Africa in the first paragraph line 70. | Thank you very much for the suggestion. Although the focus of the study was SSA, the global perspective of HIV prevalence from UNAIDs has been added: it now reads: “*Despite a 39% reduction in new HIV infections since 2010, with infections dropping from 2.1 million to 1.3 million in 2023, this figure still falls significantly short of the global goal of fewer than 370,000 new infections by 2025 [1-2].* ” | Pg 3 LN 76-79 |
|  | I would suggest quoting some figures on actual HIV prevalence rates reported in other studies in the first paragraph, to highlight the magnitude of the condition among taxi drivers. | Thank you very much for the suggestion. In the introduction, we did not provide actual HIV prevalence rates of taxi drivers and motorcyclists in SSA to avoid pre-empting the findings of our study, since our study focuses on providing the actual prevalences. Instead, we generally provided how much transport workers contribute to HIV incidence. We believe this is sufficient to explain the magnitude of the problem in this study group. |  |
| 7 | A justification on why the study focused only on Sub-Saharan Africa is needed. Taxi drivers in others parts of the world are equally at risk of HIV. A global perspective would have made the evidence produced in this study more stronger. | Thank you very much for the comment. Indeed, taxi drivers in other parts of the world are equally at risk of HIV. SSA is unique given the high HIV incidence rates, morbidity, and mortality compared with other world regions, and these are implicitly explained in the first paragraph of the introduction.  Originally, we wanted to focus our study on capturing the global perspective; however, on searching, we found that most research studies were conducted in SSA. Only 2-3 studies that met the inclusion criteria were conducted outside the SSA region. So, we realized that our findings may not represent the global perspective, which is how we narrowed down to SSA. We recognize this as a study weakness and included the following sentence under the strengths and weaknesses of the study: “Additionally, due to a narrow focus, o*ur study findings may not be generalizable to taxi drivers/ motorcyclists outside the SSA region.”* | Pg 23 LN 620-622 |
|  | **Methodology:** |  |  |
| 8 | Line 129: From inception to 2024; State a specific year. ‘Inception’ may not be very clear on which year you are reffering to. | Thank you very much for the suggestion. This has been incorporated. The inception period has been noted as January 2000 | Pg 2 LN 41, Pg 5 LN 138 |
| 9 | Line 167: Cite the SPSS software used in the analysis. | Thank you very much for the suggestion. We used the SPSS software version 20, and this has been cited in brackets. | Pg 7 LN 186 |
| 10 | Line 168: Elaborate on the qualitative data highlighted here. How did you get these data from the included studies? Did you pick the quotes reported in the studies? The approach used in getting the qualitative data is not very clear. | Thank you very much for the suggestion. We extracted the study findings and their corresponding quotation and inserted them in a Microsoft template for comprehensive reading. This sentence has been inserted in the manuscript. | Pg 7 LN 188-189 |
|  | **Results:** |  |  |
| 11 | Highlight how many studies were identified from the initial search of each individual database. | Thank you very much for the suggestion. The number of studies identified on the initial search of each database has been added to Figure 1 under records identified through database searching as:  PubMed (n=21), Google Scholar (n=54), HINARI (n=11), CINAHL (n=02), African Index Medicus (n=05), Web of Science (n=01), Scopus (n=02), and EMBASE (n=01). ” | See figure 1 |
|  | **Discussion** |  |  |
| 12 | Discuss to acknowledge how the variation in the periods of study publication may have affected the overall quality of your study. Also the Limitations section should take into consideration how studies published in 2005 may differ from those published in 2024, and how this affects the overall evidence reported here. | Thank you very much for the suggestion. This limitation has been acknowledged in this paper. The following statement has been added to the paper: “*The durations of study publication also varied, meaning that research released in 2005 could be different from that published in 2024, potentially resulting in inconsistencies and ultimately bias within the findings and conclusions*.” | Pg 23 LN 621-622 |
|  | **Conclusion:** |  |  |
| 13 | Quote the actual ‘high’ prevalence that you identified from your study. High is relative not very specific. Also, quote the HIV prevalence in the general adult population for a more clear comparison with that of the taxi-drivers. | Thank you very much for the suggestion. We meant it was very high compared with the global general adult population. We have clarified the statement and added the general adult population prevalence. | Pg 23 LN 628-630 |
| 14 | Line 603-604; highlight a few of the behavioral, psychosocial, and sociodemographic risk factors identified in this study. | Thank you very much for the suggestion. We have added a few examples of the behavioral, psychosocial, and sociodemographic risk factors. | Pg 23 LN 630-631 |
|  | **Reviewer #3:** |  |  |
| 1 | The manuscript titled "HIV prevalence, risk factors for infection, use of prevention methods, and interventions among taxi drivers and commercial motorcyclists in sub-Saharan Africa: A scoping review" addresses a significant public health concern by exploring the various aspects of HIV in these vulnerable populations. The research presented is robust and highly relevant, highlighting crucial issues that can help inform policies and interventions aimed at reversing HIV trends in sub-Saharan Africa. However, there are areas that need clarification, including terminology consistency, the categorization of studies, and alignment between statements and data presented. Overall, the authors have made a commendable contribution to the field, and with some revisions, the manuscript can provide even more impactful insights. | Thank you very much for the compliment |  |
| 2 | Thank you for the assignment to review the manuscript titled "HIV prevalence, risk factors for infection, use of prevention methods, and interventions among taxi drivers and commercial motorcyclists in sub-Saharan Africa: A scoping review." I appreciate the opportunity to contribute to this important research.  I want to thank the authors for their valuable work. The manuscript touches on a significant public health concern, and the research presented is robust and highly relevant. It addresses essential issues that are crucial for reversing HIV trends in SSA. | Thank you very much for the compliment |  |
| 3 | **Abstract:**  Replace the word “attributable to” with “associated with” since HIV infection may be associated with psychological issues like stigma but not attributable. | Thank you very much for the suggestion. We have changed the word from attributable to associated. | Pg 2 LN 50 |
| 4 | **Table 1: Search strategy**  The researcher included the term incidence and rate but the study is reporting prevalence this is confusing, kindly harmonize for clarity. Since you are also mentioning incidence in line 235 I suggest including it in the aim statement besides prevalence | Thank you very much for the suggestion. Originally, we were also interested in the HIV incidence, but we did not find it. Providing it in the search strategy and aims statement, yet we did not find any article that may be misleading. So we deleted it in the final search strategy. See table 1 again | Pg 6 LN 152 |
| 5 | line 136-138: This review included studies conducted among taxi drivers and commercial motorcyclists in SSA that reported HIV prevalence, HIV risk factors, use of HIV prevention services, or interventions that directly or indirectly reduced HIV.-Does this mean that studies where there was no reduction in HIV were excluded? | Thank you very much for the suggestion. We meant intervention that reduced HIV, but not the results of that intervention. The statement has been edited and the phrase “that directly or indirectly reduced HIV” has been deleted. The new statement reads, “*This review included studies conducted among taxi drivers and commercial motorcyclists in SSA that reported HIV prevalence, HIV risk factors, use of HIV prevention services, or HIV interventions.* ” | Pg 6 LN 155-156 |
| 6 | line 152: remove the word by after the word confirmed to enhance clarity | Thank you very much for the suggestion. The word “by” has been removed | Pg 7 LN 172 |
| 7 | line 199: Nearly half (n=11) were published within the last 5 years- You would want to specify this period – it may be confusion for audience reading this 10 years later. | Thank you very much for the suggestion. The period has been confirmed to be between 2019 and 2024 | Pg 8 LN 208 |
| 8 | lines 199-201: Most studies were conducted in the East African region (Figure 2A), with Uganda (n=8) being the largest contributor, followed by Nigeria (n=6) and South Africa (n=5). This sentence reads as if Uganda , Nigeria and South Africa are all from the East African region. | Thank you very much for the suggestion. The sentence has been broken into two for clarity. They now read as “*Most studies were conducted in the East African region (Figure 2A). Across countries, most studies were conducted in Uganda (n=8), followed by Nigeria (n=6) and South Africa (n=5).”* | Pg 8 LN 209-210 |
| 9 | line 203**:** are intervention studies not quantitative. You may want to rephrase this sentence and revise table 2 also. | Thank you very much for the suggestion. Interventional studies are indeed quantitative. Although we wanted to emphasize the type of design. We have adjusted the sentence for clarity. It now reads: “*The majority of the studies were quantitative and used cross-sectional (n=17) and interventional designs (n=2), and five were qualitative (cross-sectional designs). ”* | Pg 8 LN 210-212 |
| 10 | table 2 : Nyanzi et al. wrongly listed as qualitative study yet it is mixed methods, Potgieter et al. wrongly listed as qualitative study yet it is quantitative. | Thank you for the comment. On a closer look at Nyanzi et al., we still see it as qualitative. Although their sample size looks larger, the methods and presentation of the results are qualitative. Whereas for Potgieter et al. their study used mixed methods, we have made this adjustment in Table 2. We have also ensured that all studies that are mixed are labeled so. | Pg 9 LN 227 |
| 11 | line 231: replace the word or with the word and then add the word respectively after the word partners. | Thank you very much for the suggestion. The sentence was edited and the word respectively has been added at the end of the sentence | Pg 10 LN 248-249 |
| 12 | Line 218: is not concurring with information in figure 2B. Did some studies use more than one method? If so then this should be reported for easier interpretation of figure 2B | Thank you very much for the comment. Three studies indeed reported the HIV prevalence based on standard HIV screening and diagnosis. Unigold was reported by all three studies. Whereas determine, SDbioline, and scorecard were reported by each of the three studies in addition to Unigold. Thus, the 3 studies reported the use of more than one method. This clarity has been inserted beside the Figure 3 caption. “It is important to note that the three studies reported the use of more than one HIV determination method. Unigold was reported by all three studies. Whereas Determine, SDbioline, and scorecard were reported by each of the three studies in addition to Unigold.” | Pg 10-11 LN 256-259 |
| 13 | Line 312 to 315: Notably, no study estimated the magnitude of the risk of HIV associated with drinking alcohol, cigarette smoking, chewing khat, or using other drugs (e.g., chewable tobacco or cocaine) among taxi drivers and commercial motorcyclists. This sentence contradicts the information provided previously, Furthermore, Challe et al. reported 4.5 times odd disease among those drinking alcohol | Thank you for the comment. This mistake has been corrected, alcohol has been eliminated from the sentence, and now it reads *“Notably, no study estimated the magnitude of the risk of HIV associated with cigarette smoking, chewing khat, or using other drugs (e.g., chewable tobacco or cocaine) among taxi drivers and commercial motorcyclists. ”*  Above all, the sentence, which quotes Challe et al., has been edited: “*A high prevalence of HIV among commercial motorcyclists in Tanzania (3.9%) was found among those consuming alcohol, and they had 4.5 times the odds of acquiring HIV ”.* | Pg 13 LN 334  Pg 13 LN 325 |
